# The Role of the Lactadherin in Promoting Intestinal DCs Development In Vivo and Vitro

**DOI:** 10.1155/2010/357541

**Published:** 2010-04-06

**Authors:** Yi-Jun Zhou, Juan Gao, Hua-Mei Yang, Xiang-Liang Yuan, Tong-Xin Chen, Zhen-Juan He

**Affiliations:** ^1^Department of Pediatrics, Xinhua Hospital Affiliated to Medical Collage, Shanghai Jiaotong University, Shanghai 200092, China; ^2^Department of Pediatrics, Shanghai Sixth People's Hospital Affiliated to Medical College, Shanghai Jiaotong University, Shanghai 200233, China; ^3^Department of Laboratory Medicine, Xinhua Hospital Affiliated to Medical Collage, Shanghai Jiaotong University, Shanghai 200092, China

## Abstract

Lactadherin, as one of the immune components in the breast milk, might play a role in the intestinal immune system of newborn. Therefore, we investigated the effect of lactadherin-feeding in early time on the development of intestinal immune system compared with naturally rearing and artificially rearing (non-lactadherin). In the present study, we observed that the Peyer's Patches (PP) from the pups of artificially reared group with lactadherin added were characterized by an excess of OX62^+^CD4^+^SIRP^+^ DC cells and a higher expression of CD3^+^CD4^+^CD25^+^T cells. Additionally, this study also demonstrated that IL-10 production was dramatically increased when lactadherin was present in culture medium compared with lactadherin-absent culture. These results suggested that lactadherin could adjust intestinal DCs activity, induce CD3^+^CD4^+^CD25^+^T cell differentiation, and enhance IL-10 production.

## 1. Introduction

A relationship between breast-feeding and infant health particularly the health of the gastrointestinal tract had been recorded periodically for thousands of years across many disparate civilizations [1]. As early as in 1934, a report on 20,000 mother-infant dyads in the United States found that morbidity or mortality caused by enteric disease was several times higher in nonbreast-fed infants than in breast-fed infants [2]. These differences suggest that human milk has protective activities which are lacked in even the best artificial formulas. Recent reports associate artificial feeding of neonates with subsequent chronic diseases in later life, especially those with an immune component [3]. The mechanism has not yet been well characterized, but it could be contributed by three major phenomena. First, Human milk accelerates maturation of the gut barrier function while formula does not [4–6]. Second, the neonate may be protected from pathogenic insults during this vulnerable period by bioactive components of human milk [7]. Third, human milk components could actively adjust early immune reactions or stimulate the immune response system by activating immune factors. 

When the infant is exposed to a novel enteric pathogen, the pathogen will be presented to the dendritic cells (DCs) to subsequence T lymphocytes activity and B lymphocytes stimulation either locally in the Peyer's Patch (PP) or after migration to the mesenteric or other lymph nodes [8]. 

Nutrition may be the source of the immune system tolerant food antigens. Nutrients might modulate immune maturation and responses, and provide factors that influence intestinal flora. Through these mechanisms, it is possible that nutrition in early life might affect later immune competence, the ability to mount an appropriate immune response upon infection, the ability to develop a tolerogenic response to “self” and to benign environmental antigens, and the development of immunologic disorders [9]. However, accurate components in human milk that accelerated the evolution of the intestinal immune system in infants remain unknown. 

Lactadherin is an MW 47,000 glycoprotein in milk fat globules. It has also been known as PAS-6/7 which indicates two glycosylation variants [10], bovine-associated mucoprotein, BA-46, P47, and MFG-E8 [11]. Lactadherin has a domain structure of EGF1–EGF2–C1–C2. EGF-like domain indicates epidermal growth factor homology domain. And the C-terminal domain shares homology with the discoidin family including the lipid-binding domains of blood coagulation factor VIII and factor V [10]. The second EGF-like domain displays an Arg–gly–Asp motif [12], which binds to the av*β*5 and av*β*3 integrins [10, 13–16]. And a variety of immune-related cells such as, macrophages and DCs have the corresponding integrin receptors on the cell surface. 

It was reported that the levels of lactadherin in 41 human milk samples of 20 mothers were 93 ± 10 *μ*g/mL [17]. The amount of lactadherin was significantly higher in early milk samples (<15 d postpartum) than in later milk samples (15–90 d postpartum). Significant amounts of lactadherin were found in almost all gastric aspirates of human milk-fed infants, even 4 hours after feeding (23.2 ± 4.4 *μ*g/mL). Western blot analysis demonstrated that the immunoreactive lactadherin in the milk-fed gastric aspirates had the expected native molecular weights. There are few studies that have evaluated the role of lactadherin in the intestine immune system, which adjusts early immune reactions and stimulates the immune response system.

Our aim in this study was to investigate the role of the lactadherin in the intestine immune system by comparing among breast-fed rats, artificial-fed rats, and lactadherin-rich-fed rats. We performed artificial rearing during the suckling period with a refined formula that was made to resemble rat milk. We compared in detail the development of intestine system during suckling and weaning among the three groups.

## 2. Materials and Methods

### 2.1. Animals

SD rats were purchased from Shanghai Laboratory Animal Center (Chinese Academy of Sciences, Shanghai, China). All rats were held in the sterile plastic cages in temperature- and humidity-controlled animal rooms under a 12-hour light/dark cycle condition. Rats were fed a standard diet (rodent rat chow) ad libitum with free access to tap water. All procedures were approved by the Institutional Animal Care Committee. Three days after birth, the rat pups were weighed and randomly assigned to three groups. In the first group, 10 rat pups were reared by one of their mothers throughout the entire suckling period (breast milk-reared group, BR group). In the second group, an intragastric cannula was implanted through esophageal at the 7th day after birth using the method of Hall [18], and the rat pups were then artificially reared the formula milk without lactadherin (artificially-reared group, AR group) till the suckling period. In the third group, the same surgery was performed on the rat pups at the same day as in AR group. The rat pups were then artificially reared the formula milk with lactadherin of 100 *μ*g/ml (artificially-reared group with lactadherin added, LR group) till the suckling period, and then the cannula was removed immediately. The pups were subsequently returned to one of their mothers and reared with breast milk throughout the suckling period.

### 2.2. Artificial Rearing of Rats

The milk formula used for the AR rat pups was prepared by the method of Kanno et al. [19]under aseptic conditions, and its composition is shown in Table 1. Kanno et al. [19] prepared a refined formula, the composition of which closely resembled that of rat milk. The formula was dispensed into 50-mL sterilized polypropylene bottles, and stored frozen at 40°C. The osmotic pressure and pH of the formula milk were 343 and 6.5, respectively. The frozen formula was sterilized by gamma ray irradiation (30 kGy).

The volume of formula administered was adjusted daily according to the growth of the AR rat pups in comparison with the MR rat pups and was increased from 2.7 mL/d on day 4 to 8.2 mL/d on day 21. The rat pups were excluded when the cannula fell off during artificial rearing.

### 2.3. Reagents

Mouse antirat OX62:RPE (clone OX-62), mouse antirat CD172a:FITC (clone OX-41), mouse anti rat CD4:APC (clone W3/25), mouse antirat CD3:FITC (clone KT3), mouse antirat CD4:PE-Cy5 (clone RM4-5), mouse antirat CD8-APC (clone OX-8), and mouse antirat CD25:PE (clone IF4/OX-39/OX-6) were purchased from Serotec (Raleigh, NC, USA). 

ELISA kits of IL-10 (clone no. 31052) and IL-12 (P70 clone no. 127107) were purchased from BD Biosciences (San Jose, CA).

### 2.4. Tissue Samples

Rats were euthanized at postpartum age of 21 days, 35 days, 49 days, 63 days, and 77 days. Abdominal cavities were opened by horizontal incision along the midsection and guts were excised. Central ileum tissue samples (0.5 cm) were taken.

### 2.5. Flow Cytometry

PP tissue samples were washed 3 times in cold PBS. They were then cut into small segments and placed in cold PBS (4°C). After centrifugation at 800 rpm for 5 minutes at 4°C, the supernatant was discarded and the remaining tissues were digested with 0.75 mg/mL collagenase A for 45 minutes at 37°C with gentle rock. Undigested stoma material was removed by passing over filtration. Single-cell suspension was mashed through a cell strainer, washed once with PBS containing 2% FCS and 10 mM EDTA (staining buffer) before FACS analysis. Cells were incubated with mouse antirat OX62: RPE, mouse antirat CD172a: FITC (OX41), mouse antiratCD4: APC, mouse antirat CD3: FITC, mouse antirat CD4: PE-Cy5, mouse antirat CD8-APC, and mouse antirat CD25: PE, respectively. Staining was performed in staining buffer for 30 minutes on ice after blocking for 30 minutes. The cells were fixed in 2% paraformaldehyde before analyzed with an FACS Calibur (BD Biosciences).

### 2.6. Cell Purification and Culture

Human cord blood monocyte-derived DCs were prepared based upon standard techniques [20]. The study was approved by the Shanghai Ethical Committee of Human Genetic Resources and all subjects gave informed consent for examinations. Briefly, 30 mL of fresh human cord blood were collected in sodium heparin vacutainer collection tubes (BD Biosciences) from volunteers. The blood was diluted with PBS in an endotoxinfree bottle at a 1 : 2 ratio. CBMC were separated using Ficoll-Paque Plus at room temperature and washed with PBS. Monocytes were cultured (1 × 10^6^ per well) for 1 hour at 37°C followed by the removal of nonadherent cells; monocytes purified were >80% pure as determined by flow cytometry (data not shown). These cells (1 to 2 × 10^4^/mL) were cultured for 7 days in 6-well plates in humidified 5% CO2 at 37°C, in RPMI 1640, 10% heat-inactivated fetal calf serum (FCS), 1% glutamine, 100 U/mL penicillin, and 100 Ug/mL streptomycin, with 50 ng/mL recombinant human granulocyte-macrophage colony-stimulating factor (GM-CSF), 10 ng/mL interleukin (IL)-4, as described. Thereafter, cells were further cultured with GM-CSF and IL-4 to establish differentiation to DC phenotype, with or without lactadherin (0.5 Ug /mL) present. Medium and cytokines were renewed every 3 days. To induce maturation of DCs, 50 ng/mL tumor necrosis factor (TNF-a) was added to cultures on day 5.

### 2.7. Measurement of Cytokine Release

To detect cytokine secretion, after 7 days of culture, 10^5^ monocyte-derived DC per well supernatants were frozen at −80°C until assayed for cytokines by ELISA. Cytokine levels in supernatants were measured in triplicate with human IL-10 and human IL-12 ELISA kits.

### 2.8. Statistical Analysis

SPSS 11.0 statistical software was used for data analysis. Results were showed as mean values with standard deviation (SD). Statistical analysis was completed using one-way ANOVA. Tukey's test was used to determine the difference among groups when the ANOVA test indicated a significant effect. *P *values <.05 were considered statistically significant.

## 3. Results

There were no significant differences in either the body weight or gain in body weight throughout the suckling period and after weaning among the three groups.

### 3.1. Ontogeny Analysis of PP-DCs

**Figure 1:**
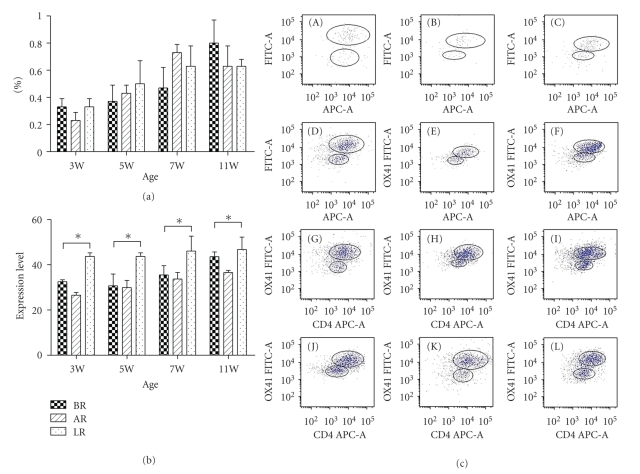
The expression of OX62^+^DCs and OX62^+^CD4^+^SIRP^+^DCs of each groups at various development stages (Mean ± SD). (a) The expression level of OX62^+^DCs of each group at different stages. (b) The expression level of OX62^+^CD4^+^SIRP^+^ DCs of each group at different stages. (c) Single-cell suspensions of the total PP-DCs in rats were identified by OX62. The difference of OX62^+^DC among groups at different development stages was not significant. (*F* = 3.0, 0.587, 3.267, and 1.471, resp.; *P* > .05). Significant growth occurred in LR group for the number of OX62^+^CD4^+^SIRP^+^DCs at age week 3. Levels of OX62^+^CD4^+^SIRP^+^DC subsets at every age were highly significant in LR group and BR group compared with AR group. Furthermore, the positive cell numbers were higher in LR group than in BR group. The positive cell numbers kept stable in LR group at various ages while the positive cells number increased with age in the other two groups. Significant numbers of OX62^+^CD4^+^SIRP^+^DCs were found in LR and BR group at various ages (Figures 1(b) and 1(c)) compared with AR group (A: BR at W3, B: AR at W3, C: LR at W3, D: BR at W5, E: AR at W5, F: LR at W5, G: BR at W7, H: AR at W7, I: LR at W7, J: BR at W11, K: AR at W11, L: LR at W11). *P < .05.

Figure 1 and Table 2 showed the development of DCs in the PPs of MR, AR, and LR rat pups during suckling and after weaning. We observed age-dependent changes of DCs in three groups. Single-cell suspensions of the total PP-DCs in rats were identified by OX62 staining. As shown in Figure 1(a), the expression of OX62^+^DC (i.e., the total PP-DC) increased with age in all three groups. However, no significant difference of OX62^+^DC expression among the three groups was observed (*F* = 3.0, 0.587, 3. 267, 1.471; *P* > .05, Table 2), regardless of the development stages. 

 With containing two populations distinguished by coexpression of CD4 and SIRP*α* or neither, significant numbers of OX62^+^CD4^+^SIRP^+^DCs were detected in LR group as early as 3 weeks of age. Furthermore, the OX62^+^CD4^+^SIRP^+^DCs were higher in LR group than in BR group. The OX62^+^CD4^+^SIRP^+^DCs kept stable in LR group while the positive cells number increased with age in the other 2 groups. Significant numbers of OX62^+^CD4^+^SIRP^+^DCs were observed in LR and BR group at various ages (Table 3, Figures 1(b) and 1(c)) compared with AR group.

### 3.2. Ontogeny Analysis of PP-T-Cells

**Figure 2:**
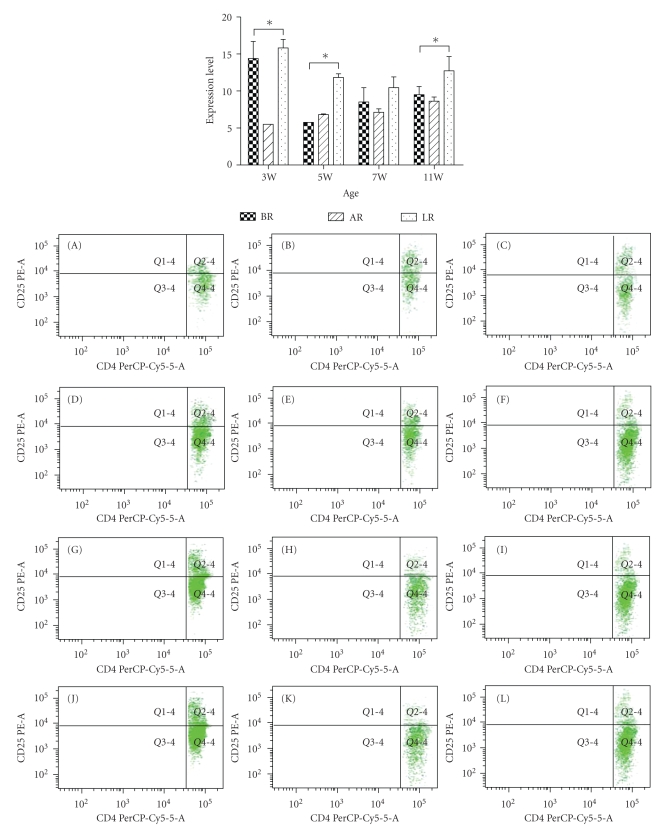
Flow cytometric analyses of CD3^+^CD4^+^CD25^+^T-cells in PPs of each group in different development stages (Mean ± SD). In order to investigate T cells proliferation and differentiation in three differently fed groups, we examined the phenotype of CD3^+^CD4^+^T cells subpopulation in the PPs by assessing the relative proportions of CD3^+^CD4^+^CD25^+^T cells. The results showed that the group of LR rats had a higher relative proportion of CD3^+^CD4^+^CD25^+^  T cells in PPs (b A: BR at W3, B: AR at W3, C: LR at W3, D: BR at W5, E: AR at W5, F: LR at W5, G: BR at W7, H: AR at W7, I: LR at W7, J: BR at W11, K:AR at W11, L: LR at W11). *P < .05.

Table 4 showed the number of the CD3^+^T cells in MR, AR, and LR rat pups. There was no difference in either value among the three groups. The CD3^+^CD4^+^ T cells (Table 5) did not differ among the MR, AR, and LR rat pups (except at age week 5), but the number of the CD3^+^CD4^+^CD25^+^ T cells in the PP was significantly higher in the LR rats than the MR and AR rats (Table 6 and Figure 2). The number of CD3^+^CD4^+^CD25^+^ T cells in the GR group was values in between those of the other two groups. The CD3^+^CD4^+^ T cells in GR group were also values in between those of the others, although there were not significantly different (except at age week 5).

**Figure 3:**
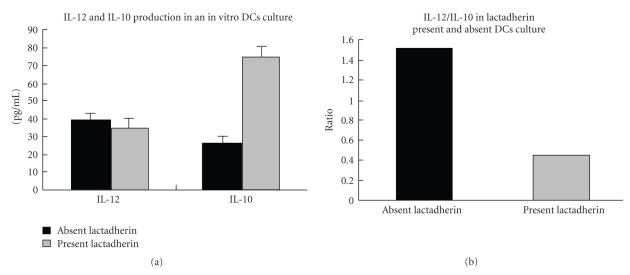
Levels of IL-12 and IL-10 production in lactadherin-present and -absent DC cultures (Means±SD). No significant differences in IL-12 production were observed with or without lactadherin present in the culture medium. However, IL-10 production was dramatically increased when lactadherin was present in the culture medium compared to when it was absent.

CD3^+^CD4^+^CD25^+^ T-cells play an important role in immunosuppression. As shown in our present study, lactadherin induces CD4^+^ T cell to differentiate CD3^+^CD4^+^CD25^+^. To address whether lactadherin could induce the cytokine secretion of PP DCs, we compared IL-10 and IL-12 production levels in an in vitro culture system. IL-12 production has no significant difference with or without lactadherin present in the culture medium. Interestingly, IL-10 production was dramatically increased when lactadherin was present in culture medium compared with that of lactadherin absent culture (Figure 3).

## 4. Discussion

Oshima et al. [21] indicated that lactadherin mRNA has previously been shown to be in the gut. However, it is still largely unknown how lactadherin functions in the intestine in vivo. Heng-Fu et al. [22] studied the role of lactadherin in maintenance and repair of murine intestine in vivo. They indicated that lactadherin played an important role in the maintenance of intestinal epithelial homeostasis. Components such as lactadherin, lactoferrin, and epidermal growth factor (EGF) are contained in maternal milk. The neonate takes these components from maternal milk, as the neonate itself cannot yet produce them in the same levels as healthy adults [23]. Spadaro et al. [24] elucidated that lactoferrin induced the maturation of human DCs and by function (cytokine production, loss of antigen internalization, and induction of T cell response). Stepankova et al. [25] elucidated that addition of EGF to diet increased the CD4^+^ T cells. PPs are typical gut-associated lymphoid tissues located along the small intestine wall and serve as the major sites for generation of immunity to intestinal antigens [26]. DCs are the most potent antigen-presenting cells for activation of further immune responses. Specifically, intestinal DCs can result in immune response limited to the intestinal mucosa without stimulating the whole immune system. Thus, intestinal DCs, one of the best characters representing the maturation and activation of intestinal mucosal immunity [27], can exist in different levels of maturation and activation that are reflected in different ways, including antigen capture and processing, effector cell activation, and cytokine networking. A subpopulation of CD4^+^ T cells that coexpress CD25 (the a-chain of IL-2R) with regulatory function has been identified in the mucosa, and has been reported to be generated by the exposure of intestinal immune system to luminal antigens. These cells appear to play an important role in the induction and maintenance of immune tolerance in the normal intestine [28]. Therefore, it can be questioned if lactadherin, one of the immune components in the breast milk, affects the immune cells compositions in PP. In order to answer this question, we designed this experiment to evaluate the effect of lactadherin function in the development of intestinal DCs and T cells. In our experiment, we developed and characterized a rat model of artificial feeding, which possesses almost all important biological similarities to the formula milk feeding human infant except for the lactadherin, as a negative control group. We observed that LG pups exhibited an excess of PP in the gut (data not shown). PPs from the pups of LR were characterized by a higher expression of OX62^+^CD4^+^SIRP^+^ DCs and CD3^+^CD4^+^CD25^+^  T cells. More experiments are needed to elucidate the dose-dependent effect of lactadherin on the development of intestinal immunity.

Nakayama et al. [29] reported that composition of microflora in artificially fed animals is different from that in mother fed animals, which might result in the diversity of T subpopulations between the differently fed animals. Indeed, we observed a significant difference between AR group and LR group pups. Whether this was due to either the pattern of bacterial colonization, cannula implantation, lactadherin, or the combination of these factors was not examined in our study. Notably, the CD3^+^CD4^+^CD25^+^ expression was higher in LR group than in AR group. As the rats of LR group and AR group received the same treatment except lactadherin, our result supported the role of lactadherin in the regulation of intestinal DCs and T cells.

Although the intestinal mucosal immune system is fully developed after birth, the actual protective function of the gut requires the antigen stimulation. It is interesting that the numbers of OX62^+^CD4^+^SIRP^+^ DC and CD3^+^CD4^+^CD25^+^ T cells are significantly (almost two times) higher in LR pups than AR pups at 3 weeks after birth, indicating that the mucosal immunity of the LR pups might be strengthened more than that of the AR pups during the suckling period. Lactadherin feeding promoted the subset of OX62^+^CD4^+^SIRP^+^ DC and CD3^+^CD4^+^CD25^+^ T cells of age week 3 rat pups; it was likely to be a consequence of weaning [30], when the sharp increase in various types of antigens occurred; lactadherin can be used as a dietary supplement to stimulate the development of intestinal immune system. In addition, even after weaning, the expressions of CD3^+^CD4^+^CD25^+^ T cells and OX62^+^CD4^+^SIRP^+^ DCs were detected at higher levels in LR pups than in MR and AR pups. There is also a good evidence of continued protection against medical condition for years after feeding in early time. The molecular mechanism underlying this process needs to be further elucidated. Another major finding of the present study is that lactadherin affected some cytokine secretion in human cord blood monocyte-derived DCs. A subpopulation of CD4+T cells that coexpresses CD25 (the a-chain of IL-2R), which possesses regulatory functions, has been identified in the mucosa and has been purported to be generated by the exposure of the intestinal immune system to luminal antigens. These cells appear to play an important role in the induction and maintenance of tolerance in the normal intestine [29]. As discussed earlier, CD3^+^ CD4^+^ CD25^+^ T-cells are potent immunosuppressors that control or prevent the development of spontaneous autoimmune diseases [30] and are involved in preventing intestinal IBD [28]. To confirm the present results, the cytokines IL-12 and IL-10, which are produced by DCs in vitro, were detected in the presence and absence of lactadherin. IL-10 is capable of inhibiting the synthesis of proinflammatory cytokines, while IL-12 is linked to autoimmunity by stimulating the production of interferon-gamma (IFN-*γ*) and tumor necrosis factor-alpha (TNF-*α*) from T and natural killer (NK) cells, and by reducing IL-4-mediated suppression of IFN-*γ*. This result showed that lactadherin enhanced DC secretion of IL-10 indicates that lactadherin might modulate intestinal allergy reactions. Additionally, the present study also demonstrated the development of a subset of DCs prior to the development of subsets of T cells. Therefore, we assume that it is likely that the development of DC subsets may drive T cells to assume distinct phenotypes and functions, although direct stimulation of T cell activity by lactadherin was ruled out. In summary, we observed that lactadherin feeding-deficient rats are characterized by a fewer number of active DCs and Tregs, reminiscent of the observations made regarding the poor immune function of formula-fed infants, which are deficient in some immune component, including lactadherin.

## 5. Conclusion

In the study, the AR rat pups matured to the same degree as MR and LR pups with respect to physical appearance. However, there were great differences in the number of OX62^+^ CD4^+^ SIRP^+^ DC and CD3+CD4+CD25+T cells among the three groups. The number of OX62^+^CD4^+^SIRP^+^ DC and CD3+CD4+CD25+T cells in LR pups showed a more similar pattern to that in the BR pups than in the AR pups. Therefore, the differences observed between the BR and AR group seemed to be the effect of the formulation of the milk rather than the effect of artificial cannulation. To our knowledge, this is the first report showing an association between lactadherin and the development of intestinal development. Our finding supports the hypothesis that lactadherin could adjust intestinal DCs activity, induce CD4^+^CD25^+^T cell differentiation, and inhibit IL-10 production. The molecular mechanism underlying this process needs to be further elucidated.

## Figures and Tables

**Table 1:** Nutrient composition and physical properties of rat's milk.

Nutrient (weight/100 mL)	Formula	Rat milk
Protein (g)	9.00	10.5 ± 1.5
Whey	3.6	3.5 ± 0.5
Casin	5.4	7.0 ± 4.8
Carbohydrate (g)	2.5	2.0 ± 1.0
Fat (g)	12	11.4 ± 4.8
Minerals (mg)		
Calcium	315	379 ± 43
Potassium	122	83 ± 19
Chloride	160	140 ± 16
Magnesium	21	22 ± 1
Vitamins		
Vitamin A (IU)	215.2	
Vitamin D3 (IU)	35	
Energy content (Kj/100mL milk)	637.0	632.0 ± 235.0
Osmolarity (mOsm/kg H_2_O)	343 ± 12	352 ± 5
pH	6.5 ± 0.1	6.5 ± 0.1

**Table 2:** The express level of the OX62^+^ intestinal DC of each group in different development stage (Mean±S) (*N* = 5).

	BR	AR	LR	*F*	*P*
3w	0.33 ± 0.06	0.23 ± 0.06	0.33 ± 0.06	3.00	.125
5w	0.37 ± 0.12	0.43 ± 0.06	0.50 ± 0.17	0.86	.471
7w	0.47 ± 0.15	0.73 ± 0.06	0.63 ± 0.15	3.27	.110
11w	0.80 ± 0.17	0.63 ± 0.15	0.63 ± 0.05	1.47	.302

**Table 3:** The express level of the OX62^+^CD4^+^SIRP^+^intestinal DC of each group in different development stages (Mean ± SD) (*N* = 5).

	BR	AR	LR	*F*	*P*
3w	32.53 ± 0.86	26.5 ± 1.3	43.7 ± 1.57	140.55	.000
5w	30.73 ± 5.17	29.93 ± 3.2	43.73 ± 1.57	13.72	.006
7w	35.50 ± 4.08	33.70 ± 2.87	46.10 ± 6.58	5.92	.038
11w	43.60 ± 2.07	36.57 ± 0.96	46.80 ± 5.49	6.97	.027

**Table 4:** The express level of the CD3^+^ T cells of each group in different development stage (Mean ± SD) (*N* = 5).

	BR	AR	LR	*F*	*P*
3w	11.23 ± 0.58	9.13 ± 3.32	19.36 ± 2.27	15.89	.004
5w	23.33 ± 3.13	19.33 ± 0.77	21.16 ± 3.11	1.79	.245
7w	20.40 ± 4.39	29.66 ± 4.04	23.93 ± 3.48	4.12	.075
11w	23.83 ± 4.56	28.76 ± 0.58	25.40 ± 3.20	1.81	.242

**Table 5:** The express level of the CD3^+^ CD4^+^ T cells of each group in different development stage (Mean ± SD) (*N* = 5).

	BR	AR	LR	*F*	*P*
3w	34.67 ± 8.04	31.73 ± 1.78	32.03 ± 2.88	0.30	.746
5w	51.36 ± 6.43	47.36 ± 7.99	63.91 ± 4.13	5.71	.041
7w	53.80 ± 12.15	61.16 ± 7.05	47.10 ± 6.75	1.83	.239
11w	52.03 ± 5.65	57.20 ± 2.55	49.66 ± 5.50	1.94	.224

**Table 6:** The express level of the CD3^+^ CD4^+^ CD25^+^ T cells of each group in different development stage (Mean ± SD) (*N* = 5).

	BR	AR	LR	*F*	*P*
3w	14.40 ± 2.30	5.50 ± 0.26	15.83 ± 1.15	41.89	.000
5w	5.76 ± 0.66	6.80 ± 0.10	11.83 ± 0.49	119.03	.000
7w	8.53 ± 1.91	7.13 ± 0.46	10.46 ± 1.45	4.20	.072
11w	9.50 ± 1.11	8.63 ± 0.57	12.73 ± 1.91	27.18	.001
